# Can Peto’s paradox be used as the null hypothesis to identify the role of evolution in natural resistance to cancer? A critical review

**DOI:** 10.1186/s12885-015-1782-z

**Published:** 2015-10-24

**Authors:** Hugo Ducasse, Beata Ujvari, Eric Solary, Marion Vittecoq, Audrey Arnal, Florence Bernex, Nelly Pirot, Dorothée Misse, François Bonhomme, François Renaud, Frédéric Thomas, Benjamin Roche

**Affiliations:** 1 MIVEGEC, UMR IRD/CNRS/UM 5290, 911 Avenue Agropolis, BP 64501, 34394 Montpellier Cedex 5, France; 2 CREEC, 911 Avenue Agropolis, BP 64501, 34394 Montpellier Cedex 5, France; 3 Université Montpellier, 163 rue Auguste Broussonnet, 34090 Montpellier, France; 4 Centre for Integrative Ecology, School of Life and Environmental Sciences, Deakin University, Waurn Ponds, Vic Australia; 5 INSERM U1009, Université Paris-Sud, Gustave Roussy, Villejuif, France; 6 Centre de Recherche de la Tour du Valat, Le Sambuc 13200 Arles, France; 7 RHEM, Réseau d’Histologie Expérimentale de Montpellier, IRCM, Institut de Recherche en Cancérologie de Montpellier, INSERM, U1194 Montpellier France, Montpellier, France; 8 ICM, 208 Avenue des Apothicaires, Montpellier, 34298 France; 9 ISEM, UMR CNRS/IRD/EPHE/UM 5554, Place Eugène Bataillon, Montpellier Cedex 5, 34095 France; 10 UMMISCO, UMI IRD/UPMC, 32 Avenue Henri Varagnat, 93143 Bondy Cedex, France

## Abstract

**Background:**

Carcinogenesis affects not only humans but almost all metazoan species. Understanding the rules driving the occurrence of cancers in the wild is currently expected to provide crucial insights into identifying how some species may have evolved efficient cancer resistance mechanisms. Recently the absence of correlation across species between cancer prevalence and body size (coined as Peto’s paradox) has attracted a lot of attention. Indeed, the disparity between this null hypothesis, where every cell is assumed to have an identical probability to undergo malignant transformation, and empirical observations is particularly important to understand, due to the fact that it could facilitate the identification of animal species that are more resistant to carcinogenesis than expected. Moreover it would open up ways to identify the selective pressures that may be involved in cancer resistance. However, Peto’s paradox relies on several questionable assumptions, complicating the interpretation of the divergence between expected and observed cancer incidences.

**Discussions:**

Here we review and challenge the different hypotheses on which this paradox relies on with the aim of identifying how this null hypothesis could be better estimated in order to provide a standard protocol to study the deviation between theoretical/theoretically predicted and observed cancer incidence. We show that due to the disproportion and restricted nature of available data on animal cancers, applying Peto’s hypotheses at species level could result in erroneous conclusions, and actually assume the existence of a paradox. Instead of using species level comparisons, we propose an organ level approach to be a more accurate test of Peto’s assumptions.

**Summary:**

The accuracy of Peto’s paradox assumptions are rarely valid and/or quantifiable, suggesting the need to reconsider the use of Peto’s paradox as a null hypothesis in identifying the influence of natural selection on cancer resistance mechanisms.

## Background

**Fig. 1:**
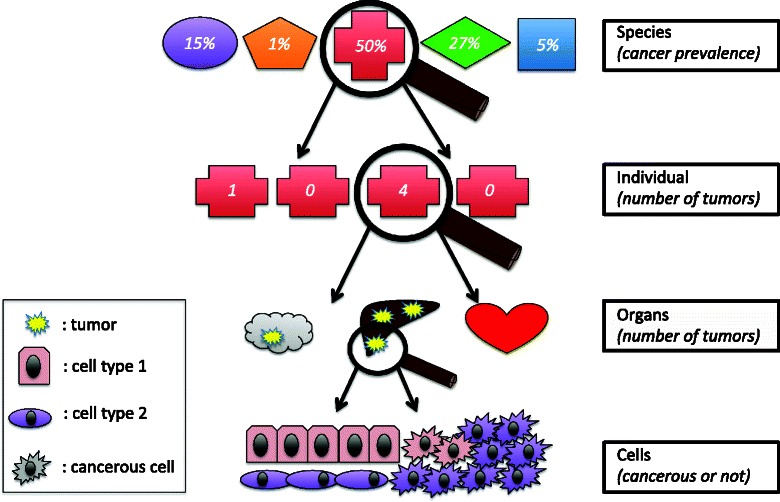
Predicted cancer risk at different scales: between different species, between individuals from the same population, between organs in an individual, and between cell types (purple cells are more at risk). From top to bottom: the different shapes represent theoretical species and variation in cancer prevalence; the red crosses represent different indivuals of the same species and the number of tumors (e.g. centenarians in a population). The third row illustrates the expected variation of tumor numbers among different organs (e.g. small intestine and large intestine). The last row shows variation in cancer risk at the cellular scale (e.g. stem cells and differentiated cells).

In the constant search for novel therapeutic strategies against cancer, identifying and understanding natural tumor suppressor mechanisms could provide promising alternative avenues; nevertheless this area of research still remains in its infancy [1–5]. While the human genome is being extensively explored for genes involved in cancer initiation or progression [6–8], analysis of cancer resistance in wildlife could also identify additional, previously overlooked, tumor suppressor mechanisms [9], and concomitantly contribute to deciphering the underlying selective forces and evolutionary processes [10]. While almost all metazoan species are affected by cancer [11–14] (Fig. 1), some animal species or individuals are more at cancer risk than others [4, 9, 15, 16], suggesting that resistance mechanisms have independently evolved in distant lineages [3, 4, 17]. For example, while rodents demonstrate a characteristically high prevalence of malignancies [18], cancer has never been observed in naked mole-rats (*Heterocephalus glaber*) [19], not even in captivity, indicating that this species has developed efficient tumor suppressor mechanisms during its evolution.

During the quest of identifying species with efficient cancer resistance, a simplistic approach can be employed [20–22]. Starting from the assumptions that carcinogenesis progresses via accumulation of mutations, and that every cell division has an identical probability to generate these mutations, a simple prediction can be drawn: large/long-lived animals should have more cancers than smaller/shorter-lived ones, due to increased number of cell divisions [3]. Actually, current evidence suggest that large/long-lived animals tend to have, on average, similar rates of cancer than small/short-lived ones [3]. A possible explanation for the absence of correlation, called Peto’s paradox [3, 22], is that evolution via natural selection may have played a significant role in shaping resistance mechanisms against malignant transformation in large/long-lived species [3, 23, 24]. Peto’s paradox postulates that animals that have evolved to be larger have also developed mechanisms to offset the increased risk of cancer. For example, some large vertebrate species have numerous copies of tumor suppressor genes (TSGs) [25], e.g. the elephant (*Loxodonta africana*) that has twelve orthologues of the human p53 gene, a key tumor suppressor fundamental to whole genome integrity. The role of natural selection is reinforced by the fact that Peto’s paradox seems not to exist at an intra-specific level, where taller individuals seem to have slightly more cancer than shorter ones [4, 16].

Although crucial to understand, this paradoxical relationship relies on a few over-simplistic assumptions (hereafter defined as Peto’s hypotheses): (i) the number of dividing-cells in an organism is strictly proportional to its size, (ii) each dividing cell has the same risk of mutation, and (iii) only mutations induce transformation to malignancy. Supporting evidence for Peto’s hypotheses is relatively scarce, mainly due to limited data on cancer prevalence in the wild [26], as well as owing to the fact that the existing evidence disproportionately focuses on certain organs and/or animal species.

The simplicity of these hypotheses cast doubts on how accurate/relevant/correct is Peto’s paradox in explaining cancer resistance, when there is clear deviation from theoretical expectations to empirical data when considering cancer prevalence in human and animal populations. Therefore, we review here Peto’s hypotheses listed above, through considering the complexity of carcinogenesis, as well as by focusing on oncogenic processes at different organismal levels: cells, organs and populations. We show that it is not only hard to accurately quantify the correctness of Peto’s assumptions, but also that the hypotheses are rarely valid, and therefore we propose to reconsider the legitimacy of Peto’s paradox. We discuss in details the potential ways to robustly assess the paradox, and argue that apart from body size, additional ecological, environmental and behavioral factors, together with the number of stem cells at the tissue/organismal levels should also be considered when assessing cancer prevalence, and attempting to identify species with resistance to cancer.

## Discussion

### Underlying hypotheses of Peto’s paradox

#### Do cell division patterns support Peto’s paradox?

The first hypothesis of Peto’s paradox postulates that large/long-lived animals have more dividing cells compared to smaller/short-lived ones. This hypothesis does not take into account the great variety of division rates within an organism where some cells could divide more frequently than others.

**Fig. 2:**
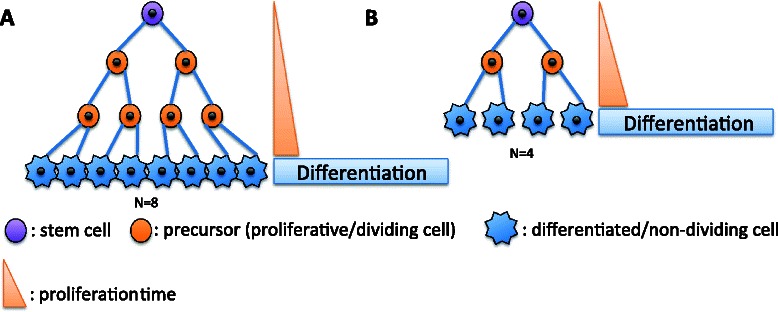
Variation in time to switch between division and differentiation results in significant cell number differences inspite of the same starting stem cell numbers. **a** Stop of proliferation and start of differentiation after three generations lead to a larger differentiated tissue mass. **b** Stop of proliferation after one generation and start of differentiation earlier than A, result in a smaller differentiated tissue mass

In many, if not most, cases, cancer may arise from transformation of stem cells [27], cells representing the first step of differentiation processes and with a great potential to divide (and/or proliferate). During the development of multicellular organisms, the obvious function of cell differentiation is to create new cell types. In adult organisms, new cell types are no longer needed or produced – but cell replacement is essential, tissues could be maintained by the self-duplication of fully mature and functional cells. Therefore, the function of ongoing, but tightly controlled cell differentiation may have evolved to protect from detrimental cell-level progression [28, 29]. With such a serial differentiation pattern, self-renewing cell populations are much more susceptible to somatic mutation, but these cells are rare and slow growing. Certain type of differentiated cells cannot initiate propagation of malignant phenotypes because they cannot divide, e.g. myocytes, adipocytes, and neurons [30]. Based on that concept, Peto’s hypothesis assumes that the number of stem cells should correlate with body mass. But the number of stem cells as well as the number of divisions have a low probability to correlate with body mass. A different number of differentiated cells may be obtained from the same number of stem cells [31] by dint of a switch between proliferation (dividing cell) and differentiation (non-dividing cell) (Fig. 2). Then, the number of divisions will not only depend on the number of stem cells, but also on the timing to switch between proliferation and differentiation (Fig. 2). The number of cells that will divide as well as the tissue turnover can be very different among organs [32], for example, in humans, the intestinal epithelium completely self-renews within ~ 5 days, while lung epithelium takes up to 6 months to be replaced [28]. Furthermore the number of stem cells is also different among organs, and this number could be involved in tumorigenesis [27]. Naively, one might think that having a larger organ requires a greater number of cells, but recent perspective papers show that differences of cell size could also be essential in determining organ size [33]. Including cell size as a parameter for the prediction of cancer risk shows that the correlation between body/organ size and cancer is weaker [33]. Furthermore, basal metabolic rate (BMR) is also decreased in larger animals compared to smaller ones (i.e. Max Kleiber allometric law [34]). Low BMR induces less oxidative stress in comparison to higher BMR [35]. Thus, larger animals could have a lower level of oxidative stress compared to smaller ones, and hence offsetting the higher cancer risk due to increased cell numbers. Indeed, a recent study by Dang, 2015 [36] support the hypothesis that metabolism can drive tumorigenesis and accounts for Peto’s paradox explanation.

#### Would transformation rates to cancer phenotypes be equivalent across different cell and tissue types?

Another assumption of Peto’s paradox, based on the fact that the rate of malignant transformation may be constant and similar across cell types, is that the mutation accumulation rate is constant among the cells. Important sources of genomic alterations are mutations, or spontaneous errors of DNA replication, [37, 38] that occur despite the existence of a wide range of mechanisms ensuring DNA repair and correct replication [39].

However, division processes – and mutation rates – may differ among cell types: for instance mutation rate has been reported to be 17 times higher in human somatic cells than in germ cells [40]. The mutation rate may differ also between organs, even though there is only limited data available on the mutational spectra of various tissue types [41]. Among differentiated cells, mutation rates of human retina cells has been estimated to be 3.7 times greater than intestinal epithelial cells, but still 1.48 times lower compared to that observed in lymphocytes [40] in which recombination events occur naturally and frequently. The rate of genetic alterations also varies across species, for example for a given organ, such as colon, mutation rates per generation is 2.14 times greater in the rat (*Rattus norvegicus*) than in the mouse (*Mus musculus*). The level of genetic variation can be intrinsic to the tissue type, e.g. the level of oxidative stress is very different across different tissue types [35]. Furthermore, mutation rates may also be affected by exposure to mutagens, especially in tissues, such as skin, respiratory and digestive epithelia, that are in direct contact with the external environment and then naturally more exposed to mutagens and radiations. Differences may also exist between similar organs in diverse animal species [40].

As suggested above, additional mechanisms, especially for lymphomas and leukemia, can increase DNA instability in specific cell types such as T and B lymphocytes, some of the key cells of the vertebrate immune system. One of the important characteristics of lymphocytes is that a specific part of their coding genome is hypermutated to generate the incredible genetic diversity necessary to recognize the plethora of foreign antigens, and hence protect the organism from a broad range of pathogens [42]. The enzymes involved in initiating the hypermutation events could potentially also increase the genomic instability of these cells and favor errors leading to lymphoid transformation [43].

#### Would carcinogenesis rely on mutations only?

The last assumption of Peto’s paradox is that a variety of somatic genomic alterations, from single nucleotide variants to larger structural aberrations (including insertions, deletions, and chromosomal translocations) can contribute to cell transformation (somatic mutation theory [44]). The genetic alterations will then be transmitted through DNA replication and cell division to the daughter cells. However, spontaneous mutations are insufficient to explain cell transformation in every situation [45–47], and cancer can potentially also arise from a variety of other mechanisms, which may vary between organs and species (Table 1).

**Table 1:** Main cancer causes apart from that mentioned in Peto’s paradox

**Genetic predisposition:** heritable mutations that confer a higher cancer risk, for instance mutations in BRCA1 and 2 genes associated with 40–60 % cumulative risk of breast cancer [104].
**Pathogens:** some infectious agents like viruses, helminthes or bacteria could also trigger tumor development. For instance, schistosomes have been shown to induce bladder cancer, Human Papilloma Virus is associated with cervical cancer or *Helicobacter pylori* (bacteria) increases the risk of stomach cancer [87].
**Pollutants:** Pesticides, smoking or electromagnetic radiation are associated with increased risk of cancer [105]. A study conducted by the American Cancer Society shows that an increase of 10 micrograms per cubic meter of fine particles in suspension would potentially cause an 8–14 % increase of lung cancer cases [106].
**Alimentation:** There is a positive correlation between obesity and cancer mortality [107]. In fact obese people secret more leptine, a hormone which *in vitro* stimulates cancer cell proliferation [108].

##### Variation in mutation numbers required to trigger tumor formation and progress

The number of genetic alterations varies largely, depending on age and tumor type, e.g. the number of genetic alterations is usually reduced in pediatric tumors such as juvenile myelomonocytic leukemia [48, 49] or acute megakaryoblastic leukemia [50] while being the highest in lung cancers induced by smoking [51] and melanomas induced by UV [52, 53]. The genomic signature of tumor cells (established based on the nature, localization and number of genomic alterations identified in the affected cells) informs about the factors that have promoted and contributed to the malignant transformation (ageing versus toxic exposure versus genetic predisposition etc…) [54–57]. Solid tumors usually carry more genomic alterations than hematological malignancies [58–60].

Furthermore, the functional consequences of a given mutation are highly variable, depending on its nature and localization in the genome. Those that have the most striking effects are those that activate a proto-oncogene (e.g. genes involved in cancer initiation/progression) or inactivate a tumor suppressor gene (e.g. genes that allow apoptosis or stop cell-cycle). A single nucleotide change can be sufficient to transform a proto-oncogene into an oncogene that induces cell transformation, whereas an inhibiting mutation must affect the two alleles of a tumor suppressor gene to favor transformation [61].

##### Epigenetic factors

In addition, a growing number of studies show that epigenetic stochasticity can act as driving force of carcinogenesis, via regulating the inhibition of tumor suppressor genes [62] as well as the activation of proto-oncogenes [63]. Since epigenetic stochasticity is not correlated to body size, it may introduce background noise when testing Peto’s paradox. Furthermore, since environmental factors (e.g. species ecology, habitat, resource availability) can significantly influence transgenerational epigenetic modifications, it can thus be important to consider both consistent and stochastic (e.g. oil spills, famine, extreme climate parameters) environmental changes across generations in order to decipher their contribution to tumor formation [62].

##### Tumor microenvironment

In addition to spontaneous mutation and epigenetic mechanisms, it is also increasingly recognized that tissue organization plays a major role in the development of malignant phenotypes (tissue organization field theory) [44]. This theory relies on the fact that cancer cells can proliferate only within a suitable microenvironment [1, 64], a particular tissue environment with specific conditions, e.g. low pH and/or oxygen concentrations [65]. Generally, normal tissue homeostasis and architecture inhibit progression of cancer, but changes in the microenvironment can shift the balance of these signals to a cancer permissive state. Tumor development, progression and metastasis are strongly dependent on the microenvironmental conditions met by cancer cells [1]. Tumor ecosystems consist of non-malignant normal cells (fibroblasts, immune cells and cells that comprise the blood vessels) and heterogeneous cancer cells, as well as their cellular products supporting cancer cell growth. Interactions between cancer cells and the surrounding microenvironment are constant, and bidirectional. Tumors can influence the microenvironment by releasing extracellular signals, promoting tumor angiogenesis and inducing peripheral immune tolerance. In return, the immune cells in the microenvironment influence the growth and evolution of cancerous cells (e.g. immune-editing [66]).

Animal models have demonstrated that alterations in the tissue microenvironment can promote the emergence of clonal malignancies, e.g. mutation in Dicer genes (involved in RNA interference) generated in the bone marrow microenvironment can promote the emergence of a leukemic clone [67, 68]. Lastly, the recent success of immunotherapeutic strategies demonstrates that suppression of the anticancer immune response is required for a tumor to emerge [69]. Therefore, even if cells have enough mutations to initiate carcinogenesis, malignant cells won’t develop without a permissive cancer niche and immune system, which will be then dependent of the tissue, the organ, and the species [65].

Thus, a Darwinian evolution of host factors relating to resistance may be more relevant for an explanation of Peto’s paradox, than carcinogenesis parameters such as cell divisions or stem cell number.

### Peto’s paradox at the population level: artifact or reality?

#### Sampling bias

Assessment of Peto’s paradox [3, 4, 12, 20] relies on cancer incidence measured over very few species, i.e., dog (*Canis lupus domesticus*), mouse (*Mus musculus*), beluga (*Delphinapterus leucas*) and humans [12], covering a small gradient of the possible body mass. Another possible bias, when assessing this paradox, is that the detection of cancer relates only to the presence of macroscopic tumors, and thus neglects the precancerous lesions or the microscopic tumors of vital organs. Thus, due to the bias of studied species, current datasets are definitely lacking power to determine the exact relationship between body mass and cancer incidence [70].

Additionally, other sampling biases may also explain the lack of relationship between body mass and cancer prevalence. Of particular concern is that research so far has predominantly relied on domesticated and laboratory animals when attempting to establish the correlation. While the role of artificial selection for certain traits has been recognized [71], it seems to also apply to the emergence of cancer phenotypes. Anthropogenic selection (including domestication and breeding for particular traits in the laboratory) could have additionally led to artificial selection for cancer resistance or susceptibility. Therefore, laboratory and domesticated species, e.g., mice and dogs, could have cancer incidences different from wildlife species because of an inadvertent selection of traits involved directly or indirectly with carcinogenesis.

#### Environmental factors triggering the development of cancer phenotypes

Inter-species comparison can be challenging and misleading due to the fact that cancer initiating factors are probably not the same between different species. Indeed, comparison between human and other species could be biased by different levels and types of exposure to environmental and behavioral factors, including pollution, abundant and excess food supply, and frequent contact with mutagens [72, 73]. For instance, while there is no significant difference between body size of roe deer (*Capreolus capreolus*) and humans (on a logarithmic scale), cancer incidence is much higher in humans (20 % versus 2 % for roe deer) [74–76]. These different incidences could be explained by physiological parameters, but also by a differential exposure to mutagens. Furthermore, human cancers have been studied more extensively and on a broader scale than the ones observed in wildlife, i.e., roe deer. Similarly, although extensive data is available on relatively high cancer prevalence in Belugas (27 %), these numbers originate from a pod of whales living in a polluted environment, suggesting that cancer prevalence could also be overestimated for this species, just like for humans [20]. For humans, the way of life may be critically important, for instance low concordance rate for leukemia in identical twins (5 %) suggests that additional postnatal exposure should influence leukemia development [77].

Comparing animal species occupying different trophic levels can also jeopardize the identification of animal species with resistance to cancer. For instance, mutation is also driven by cellular proliferation after injuries. Therefore, species with high injury rate from predators or aggressors should have evolved faster wound healing/tissue regeneration [78, 79], which could concomitantly increase the number of malignant transformations due to increased level of cell proliferation being associated with growth factors induced in tissue regeneration [80]. Furthermore, occupying different ecological niches can also contribute to various levels of cancer prevalence. For example, natural habitats of large mammals, such as elephants or beluga whales (except the aforementioned pod of whales), are significantly less polluted than the habitat of benthic organisms that are more exposed to contaminated sediments [81].

It is recognized that for many species longevity is highly correlated with size [82], but there are also noticeable exceptions, for instance the naked-mole rat that displays a maximum lifespan of 28.3 years for a mass of 35 g (in contrast to a similar size *Mus musculus* with a maximum lifespan of 3.5 years) [83]. Due to a long-lived organism potentially accumulating more mutations during its life [45, 84], it is expected that selection will favor cancer resistance in small, but long-lived species to circumvent the higher risk of cancer due to mutation accumulation (e.g. naked mole rate [85, 86]). Thus, for species displaying an atypical relationship between size and longevity, cancer resistance pattern will not follow the traditional prediction derived from Peto’s paradox.

Finally, increasing number of studies suggest that at least some cancers may have infectious origins [87]. The number of pathogen known to be associated with cancer in wildlife has also been on the rise, for example woodchucks (*Marmota monax*) suffering from hepatocarcinomas originating from hepatitis virus infections [88] and marine turtles succumbing to fibropapillomatosis also caused by viruses [89]. Several studies have focused on comparative analysis of parasite communities, and on the determining factors of parasite species richness, heterogeneity and densities [90–92]. A relationship between body size and parasite species richness is thus possible, for example it has been shown that endogenous retroviruses abundance negatively correlates with body mass [93].

## Summary

The disproportion and restricted nature of available data can make a paradox seemingly exist despite the actual lack of support for it. In this review, we have shown that the hypotheses behind Peto’s paradox are rarely supported by evidence, and therefore we question the relevance of using this paradox as a null hypothesis to identify selective pressures shaping cancer resistance mechanisms. Nevertheless, we emphasize that deciphering the relationship between ecological and behavioral parameters of animal species and cancer incidence can be essential and important to the identification of species which have evolved effective tumor resistance mechanisms. In addition, given the recent paper by Tomasetti and Vogelstein 2015 [27], we propose here that future research on Peto’s paradox should be envisaged from the number of stem cells per individuals/species rather than on the body size which seems to be an unreliable surrogate.

In this review, we have shown that having more cells does not necessarily mean increased number of malignant transformations, because different cell types have different division rates, and DNA mutations can accumulate at different frequencies through various mechanisms. Thus, each organ and each species should have different cancer prevalence. If organ size and tissue environment were equivalent across species, then the shortcomings of Peto’s hypotheses should not matter, and Peto’s paradox would remain valid. However, reality of animal species is obviously more complex due to physiological, ecological and evolutionary constraints of organisms.

Although Peto’s assumptions are not satisfied at cellular level, it is still possible to test Peto’s paradox across species by considering cancer in each organ separately. Due to individual organs having similar cellular structure and micro-environment across different species, a cross-species comparison of given organs would definitely be more informative, and would allow more rigorous and valid testing of Peto’s paradox.

However, the physiology of the organ should be considered carefully. For instance, only focusing on a digestive organ could lead to biased predictions due to the size of digestive organs being strongly influenced by diet (e.g. carnivore vs herbivore [94–97]), the digestive tract of herbivores will be larger compared to carnivores, to allow an optimal digestion of cellulose [98].

Another possibility is to focus on the genome size. In fact, the variation observed in genome size across species could provide the foundations to the principles of Peto’s paradox (Animal Genome Size Database [99]): cancer incidence should be positively correlated with genome size rather than body size. A bigger genome should induce higher probability of mistakes in DNA replication during cell division, leading to higher risk of mutation and concomitantly to cancer. It has been proposed that extremely large genomes (like those of certain tree species) are an adaptation to withstand somatic mutations over the long haul, because of the mutagenic effects of pollutants, radiations or transposable elements are diluted [100] inside non-coding (and hence not harmful) junk DNA.

The philosophy of Peto’s paradox can be nevertheless applied at different scales. Indeed, within a given species, since each organ has its own tumor prevalence, one could propose the existence of higher cancer risk for larger organs [101] or, if Peto’s paradox exists at organ level, a lower cancer risk could be associated with resistance mechanisms driven by gene expression variations. For instance, pancreas size is conditioned by the initial number of progenitor cells. Therefore, size and cell number of this organ are fixed for the rest of the life, unlike the size/cell number of liver which could increase over a lifetime [102].

If Peto’s paradox could only be applied to organs, one should however also take into account that (i) the rate of regeneration of organs can vary between individuals depending on different exposure levels to mutagens (e.g. organs involved in removal of toxic materials (kidney), or organs in direct contact with external environment (digestive organs, lung)), (ii) the different mutation rates and (iii) the connections between the organs that may influence the spread of metastases by predetermined cellular pathways [103].

## Conclusions

 According to the different factors that may bias our interpretation of Peto’s paradox, comparing cancer prevalence across different species should take into account several fundamental parameters. Given that the assumptions of Peto’s paradox are not supported by strong evidence, our review suggests alternative ways for a more robust testing of the correlation (or rather lack of it) between body size and cancer risk. First, in view of great intra-individual variability in mutation and division rates across organs, it would be more appropriate to compare cancer prevalence in each organ separately [20]. Furthermore, since environmental factors can dramatically influence carcinogenesis, the integration of these factors would be essential to the accurate estimation of cancer prevalence across species. For an effective analysis, we suggest to compare species occupying similar ecological niches, or living in habitats where environmental factors can be controlled for, such as zoos or nature reserves.

We propose that refuting Peto’s paradox is actually not the most important question to be answered. Rather, investigating the lack of correlation between body mass and cancer incidence (the foundation of Peto’s paradox) opens up the opportunities to explore and answer such important queries as to how the random appearance of malignant cells influences cancer prevalence, and whether we could identify tumor resistance mechanisms without exploring entire genomes. The required next step is thus to estimate correctly this null hypothesis in order to interpret correctly this paradoxical relationship. Even if the three Peto’s hypotheses are flawed, it is crucial to determine whether the impact of discrepancies is enough to explain the lack of correlation between cancer risk and size/longevity at the interspecific level.
